# Inhibition of Anaplastic Lymphoma Kinase (ALK) Activity Provides a Therapeutic Approach for CLTC-ALK-Positive Human Diffuse Large B Cell Lymphomas

**DOI:** 10.1371/journal.pone.0018436

**Published:** 2011-04-08

**Authors:** Leandro Cerchietti, Christine Damm-Welk, Inga Vater, Wolfram Klapper, Lana Harder, Christiane Pott, Shao Ning Yang, Alfred Reiter, Reiner Siebert, Ari Melnick, Willi Woessmann

**Affiliations:** 1 Hematology and Oncology Division, Weill Cornell Medical College, Cornell University, New York, New York, United States of America; 2 Pharmacology Department, Weill Cornell Medical College, Cornell University, New York, New York, United States of America; 3 Department of Pediatric Hematology and Oncology, Justus-Liebig University, Giessen, Germany; 4 Institute of Human Genetics, Christian-Albrechts-University Kiel and University Hospital Schleswig-Holstein, Campus Kiel, Kiel, Germany; 5 Department of Pathology, Hematopathology Section and Lymph Node Registry, University Hospital Schleswig-Holstein, Campus Kiel, Kiel, Germany; 6 2nd Medicine Department, Christian-Albrechts-University Kiel and University Hospital Schleswig-Holstein, Campus Kiel, Kiel, Germany; Wellcome Trust Centre for Stem Cell Research, United Kingdom

## Abstract

ALK positive diffuse large B-cell lymphomas (DLBCL) are a distinct lymphoma subtype associated with a poor outcome. Most of them feature a t(2;17) encoding a clathrin (CLTC)-ALK fusion protein. The contribution of deregulated ALK-activity in the pathogenesis and maintenance of these DLBCLs is not yet known. We established and characterized the first CLTC-ALK positive DLBCL cell line (LM1). LM1 formed tumors in NOD-SCID mice. The selective ALK inhibitor NVP-TAE684 inhibited growth of LM1 cells *in vitro* at nanomolar concentrations. NVP-TAE684 repressed ALK-activated signalling pathways and induced apoptosis of LM1 DLBCL cells. Inhibition of ALK-activity resulted in sustained tumor regression in the xenotransplant tumor model. These data indicate a role of CLTC-ALK in the maintenance of the malignant phenotype thereby providing a rationale therapeutic target for these otherwise refractory tumors.

## Introduction

Expression of anaplastic lymphoma kinase (ALK) fusion proteins resulting from chromosomal translocations involving chromosome band 2p23 is a hallmark of anaplastic large cell lymphomas (ALCL) [1]. The most frequently detected ALCL translocation t(2;5) encodes a nucleophosmin (NPM)-ALK fusion protein with constitutive tyrosine kinase activity which is clearly implicated in the pathogenesis of ALCL [2], [3].

Diffuse large B cell lymphomas (DLBCL) harbouring ALK fusion proteins were first described in 1997 [4]. With few exceptions these ALK-translocated DLBCLs display a fine granular cytoplasmic ALK-staining characteristic for the fusion of clathrin (CLTC) with ALK caused by the reciprocal translocation t(2;17)(p23;q23) [5], [6], [7]. These DLBCLs are further characterized by the expression of immunoglobulin light chain kappa or lambda, plasma cell associated antigens CD38 and CD138, and epithelial membrane antigen (EMA), but lack expression of CD30 antigen and many other B- and T-cell markers [4], [6], [7]. From the published case reports based on approximately 50 patients, these lymphomas seem to be associated with a poor outcome in children and adults compared to both ALK-positive ALCL and ALK-negative DLBCL when treated with current chemotherapy regimens [8], [9].

Small molecule inhibitors of the ALK kinase have recently been developed [9], [10]. However, their therapeutic potential in ALK positive DLBCL has not been studied so far in part due to the lack of representative preclinical models. We report the characterization of the first CTLC-ALK positive DLBCL cell line (LM1), the establishment of a pre-clinical model to study the role of CLTC-ALK activity in DLBCL lymphomagenesis, and demonstrate that these lymphomas display activation of ALK signalling pathways and are potently suppressed in vitro and in vivo by a selective ALK inhibitor.

## Materials and Methods

### Ethics Statement

The tissue donor was included in a protocol approved by the Institutional Review Board of the Justus-Liebig University in 1999 that included the use of biopsy material for further biological studies. In accordance, the parents of the patient gave a written informed consent that included the use of tumor material and normal bone marrow for cell banking as well as for the establishment of the tumor cell line and use of the cells for further studies. Procedures involving animals followed National Institutes of Health guidelines and were approved by and done according to guidelines of the Animal Institute Committee of the Weill Cornell College of Medicine (Antitumor Assessment Core Facility of Sloan-Kettering Institute, specific protocol ID 0803732-A).

### Cell lines and chemicals

The DLBCL cell lines Karpas422 and LM1, the ALCL cell lines SUDHL1 and Karpas299 and the BL cell line DG75 were grown in medium containing 90% RPMI and 10% FCS supplemented with antibiotics, L-glutamine and HEPES. The cell lines Karpas422, Karpas299, SUDHL1 and DG75 were obtained from the Deutsche Sammlung von Mikroorganismen und Zellkulturen (DSMZ) repository that performs authentication based on a battery of appropriate test procedures including immunotyping and genotyping. Cells were maintained in these conditions during the experiments and NVP-TAE684 (hereon TAE-684) was added from a concentrated DMSO stock solution to the 10% serum-containing culture medium. The ALK-inhibitor TAE-684 (CID16038120) was synthesized in N. Gray's laboratory [10].

### Reverse transcriptase polymerase chain reaction (RT-PCR) and sequencing

Total RNA was extracted from cell lines or frozen tumor material with Trizol reagent according to the manufacturer's instructions (Invitrogen, Carlsbad, CA). cDNA synthesis was performed with 1 µg of total RNA, random hexamers or oligo-dT and Superscript-II/III reverse transcriptase (Invitrogen). Reverse Transcriptase (RT)-PCR conditions and primers were previously described [11]. Additional primers are shown in **Table S1**. In experiments involving TAE-684, LM1 cells were treated with DMSO or TAE-684 10 nM for 12 h and the RNA isolated using RNeasy Plus kit (Qiagen) following the manufacturer instructions. cDNA was synthesized using High Capacity RNA-to-cDNA kit (Applied Biosystems). We amplified specific genes using the Fast SYBR Green conditions (initial step of 20 sec at 95 C followed by 40 cycles of 1 sec at 95 C and 20 sec at 60 C). The C_T_ value of the housekeeping gene (RPL13A) was subtracted from the correspondent genes of interest (ΔC_T_). The standard deviation of the difference was calculated from the standard deviation of the C_T_ values (duplicates). Then, the ΔC_T_ values of the TAE-684-treated cells were expressed relative to their respective DMSO-treated cells using the ΔΔC_T_ method. The folds of expression for each gene in cells treated with the drug relative to control treated cells is determined by the expression: 2^−ΔΔCT^. Results were represented as fold of expression with the standard error of the mean (SEM) for 2 series of duplicates. The CLTC-ALK specific RT-PCR fragment from frozen tumor at the time point of relapse was cloned in the PCR 2.1 TOPO vector (Invitrogen). Sequencing analysis of the CLTC-ALK plasmid was performed on an ABI PRISM 3100 (Applied Biosystems, Foster City, CA) automated sequencing analyzer using standard sequencing methods.

### Immunoblotting, phospho-array and flow cytometry

Cell lysates were prepared using 50 mM Tris pH 7.4, 150 mM NaCl and 1% NP-40 lysis buffer. Lysates for nuclear and cytoplasmatic fractions were obtained using a fractionation kit (Biovision) following the manufacturer's instructions. Protein concentrations were determined using the BCA kit (Pierce). Fifty micrograms of protein lysates were resolved by SDS-PAGE, transferred to nitrocellulose membrane, and probed with the indicated specific primary antibodies: rabbit to Akt (Cell Signaling), rabbit to STAT3 (Cell Signaling), rabbit to p44/p42 MAPK (Cell Signaling), mouse anti RPS6 (Cell Signaling), rabbit anti phosphorylated Akt (Ser473) (Cell Signaling), rabbit anti phosphorylated p44/p42 MAPK (Thr202/Tyr204) (Cell Signaling), rabbit anti phosphorylated RPS6 (Ser235/236) (Cell Signaling), rabbit anti phosphorylated STAT3 (Tyr750) (Cell Signaling) and mouse to Alk (BD Bioscience, San Jose, CA). Membranes were then incubated with a peroxidase-conjugated correspondent secondary antibody. Detection was performed using an ECL detection system. Relative levels of protein phosphorylation in LM1 cells treated with DMSO or TAE-684 10 nM for 24 h were determined using a phospho-array (Proteome Profiler Array ARY003, R&D Systems, Inc., Minneapolis, MN) following the manufacturer instructions. The scanned film image was analyzed using the ImageJ freeware (NIH, Bethesda, MD). The spot density of the proteins of interest was normalized using the spot density of the positive controls. A detailed protocol and localization of the proteins in the array can be accessed in http://www.rndsystems.com/pdf/ary003.pdf. Flow cytometry was performed with a BD FACSCalibur (BD Bioscience, San Jose, CA) using CD30-FITC and CD45-APC antibodies for surface staining and ALK-PE for intracellular staining. All antibodies were from BD Bioscience (San Jose, CA).

### IGHV mutation analysis

IGHV mutation analysis was performed by multiplex PCR using the BIOMED2 protocol. Sequences were compared with published germ line V_H_, D, and J_H_ genes using the International ImMunoGeneTics (IMGT) database (http://www.imgt.org) Mutational status was calculated as percent deviation from the closest matching germ line V_H_ segment.

### Single nucleotide polymorphism (SNP) array analysis

The Genome-Wide Human SNP Array 6.0 (Affymetrix, Santa Clara, CA) has been used according to the protocol provided by the manufacturer (Affymetrix). Microarrays were washed and stained with the Fluidics Station 450 (Affymetrix) and scanned with the GeneChip Scanner 3000 (Affymetrix) using the Command Console software (Affymetrix). The Birdseed v2 algorithm was used to genotype tumor samples. Copy number analysis, loss of heterozygosity analysis and segmentation was calculated using Genotyping Console software version 3.0.2 (Affymetrix).

### Growth inhibition determination

Cell lines were grown at their respective concentration that were sufficient to keep the untreated cells in exponential growth over the 48 h drug exposure time. We determined cell viability by using a fluorometric resazurin reduction method (CellTiter-Blue, Promega, Madison, WI) following the manufacturer's instructions. The fluorescence (560_Ex_/590_Em_) was determined using the Synergy4 microplate reader (Biotek Inc, Winooski, VT). Fluorescence was determined for six replicates per treatment condition or controls. We normalized cell viability in TAE-684-treated cells to their respective controls (DMSO). We used CompuSyn software (Biosoft, Great Shelford, Cambridge, UK) to plot the dose-effect curves and to determine the concentration of drug that inhibits 50% the growth of cell lines compared to control treated cells (GI_50_).

#### Activated STAT DNA binding assay

The DNA-binding capacity of STAT3 and STAT5a was assayed by plate-based assay (TransAM, Active Motif, Carlsbad, CA) following the manufacturer instructions. Briefly, 5×10^6^ LM1 and Karpas422 cells were treated with TAE-684 10 nM or DMSO control for 4 h. Five micrograms of cell lysates were added to wells containing pre-adsorbed STAT consensus oligonucleotides (5′-TTCCCGGAA-3′). For control treated cells the assay was performed in the absence or presence of 20 pmol of competitor oligonucleotides that contains either a wild-type or mutated STAT consensus binding site. Interferon-treated HeLa cells (5 µg per well) were used as positive controls for the assay. After incubation and washing, rabbit polyclonal anti-STAT5a or anti-STAT3 antibodies (1∶1000, Active Motif) were added to each well, followed by HPR-anti-rabbit secondary antibody (1∶1000, Active Motif). After HRP substrate addition, absorbance was read at 450 nm with a reference wavelength of 655 nm (Synergy4, Biotek, Winooski, VT). In this assay the absorbance is directly proportional to the quantity of DNA-bound transcription factor present in the sample. Experiments were carried out in triplicates. Results were expressed as arbitrary units (AU) from the mean absorbance values (450/655) with SEM.

### Cell-Cycle-Analysis

Exponentially growing LM1 and Karpas299 cells were incubated with 10 nM TAE-684 or DMSO for 4, 12 and 24 h. Cells were fixed with 70% ethanol and incubated for 2 h at 4°C. After washing with ice cold PBS the cells were incubated with 50 µg/ml RNAse A and 50 µg/ml propidium iodide at 37°C for 30 m. Cell cycle distribution was analyzed with a FACS-Calibur flow cytometer (BD Bioscience, San Jose, CA).

### Detection of apoptotic cell with Annexin-V

Distribution of apoptotic, death and viable cells were determined by using Annexin-V-PE Apoptosis detection Kit I according to the manufacturers instructions (BD Bioscience, San Jose, CA). Briefly, 4×10^5^ proliferating LM1 and Karpas299 cells were treated with DMSO or 10 nM TAE684 for 24 h After washing with PBS, cells were stained with Annexin-V-PE and 7-AAD at RT for 15 m. Cells were analysed on a FACS-Calibur with Cell Quest Pro software (BD Biosciences, San Jose, CA).

### Caspase-7 and -3 activity

The activity of caspase-7 and caspase-3 was determined using the Apo-ONE caspase 3/7 assay (Promega). Cell lines were treated with TAE-684 10 nM or control for 4 h followed by 1 h exposure to the pro-fluorescent Z-DEVD-R110 substrate. Activation of Z-DEVD-R110 by the activity of caspases 3 and 7 allows the R110 group to become intensely fluorescent (Exitation_499 nm_/Emission_521 nm_), which was measured using the Synergy4 microplate reader in four replicates. Caspase-7 and -3 activity was related to the cell number determined by CellTiter-Blue (Promega) in a multiplex assay. Results are expressed in relative fluorescent units (RFU) normalized to cell number.

### Cell proliferation

LM1 cell proliferation was determined by measuring incorporation of the nucleoside analog 5-ethynyl-2′-deoxyuridine (EdU) into newly synthesized DNA following the manufacturer instructions (Click-iT EdU, Invitrogen) with modification for suspension cells. LM1 cells were treated with DMSO or TAE-684 5, 10 and 20 nM for 1 h following incubation with EdU reagent for additional 23 h. Experiment was carried out in 4 replicates. EdU incorporation was measured by the abundance of a fluorescent product (Amplex UltraRed reagent, Invitrogen) and normalized to the viable cellular number determined by dye exclusion (ethidium bromide and orange G staining, easycount, Immunicom).

### Mice xenotransplant models

Six to eight-week old male SCID and NOD-SCID mice were purchased from the National Cancer Institute (Bethesda, MA) or from Charles River Laboratories International Inc, (Wilmington, MA). Mice were subcutaneously injected in the left flank with low-passage human LM1 and Karpas422 DLBCL cells. Tumor volume was monitored every other day using electronic digital calipers (Fisher Scientific, Hamptom, NH) in two dimensions. Tumor volume was calculated using the formula: Tumor Volume (mm^3^) = (smallest diameter^2^×largest diameter)/2. When tumors reached a palpable size (approximately 75 to 100 mm^3^), the mice were randomly assigned to different treatment arms; in consequence these experiments were all performed once tumors had fully formed in the animals. TAE-684 was dissolved in vehicle (10% pyrrolidinone, 90% PEG300, both reagents from Sigma, Saint Louis, MO) and administered by oral gavage. Mice were weighed twice a week. All mice were euthanized by cervical dislocation under anesthesia when at least 2/10 tumors reached 15 mm in any dimension that for the cell lines used corresponded approximately to 5 weeks. Directly after euthanasia, all organs and tissues underwent careful macroscopic and microscopic (hematoxylin-eosin staining) examination for signs of toxicity.

### Immunohistochemistry

Slides were stained using standard procedures using Envison reagents (Dako, Glostrup Denmark) following the manufacturer instructions. Microscopic pictures were acquired using a final 400-X magnification with an Axioscope 40 microscope (Zeiss, Jena, Germany) corresponding to a 0.5 mm picture diameter at room temperature with a Color Vision 3 camera (Soft Imaging Systems, Münster, Germany). Pictures were adjusted in respect of sharpness and brightness using Adobe Photoshop 5.0 software (Adobe Systems Inc, San Jose, CA).

## Results

### Establishment of the CLTC-ALK positive DLBCL cell line LM1

**Figure 1:**
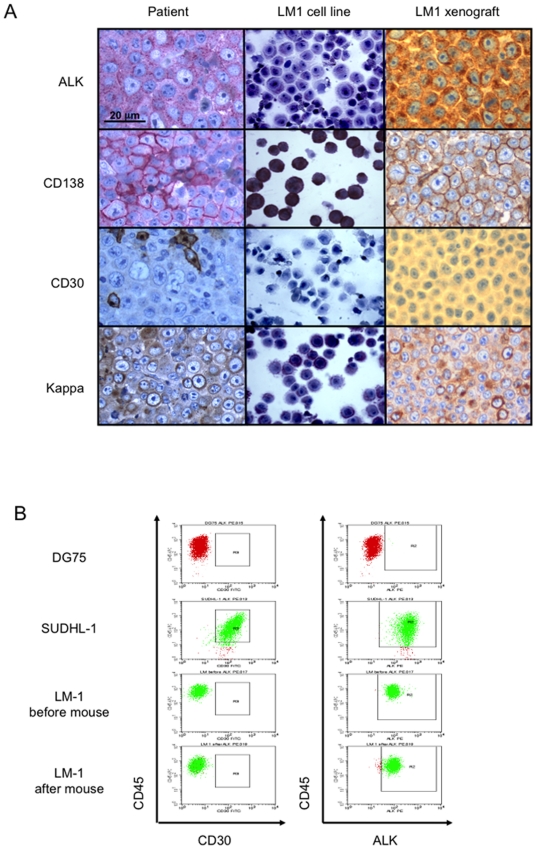
Immunophenotyping analysis of the original lymphoma and the derived cell line LM1. **A**: Determination (from top to bottom) of ALK, CD138, CD30 and Ig kappa chain expression and distribution by immunohistochemistry in the original tumor (left column), LM1 cell line grown in vitro (middle column) and LM1 xenografted tumor in NOD-SCID mice (right column). **B**: The expression of CD30 (left column) and ALK (right column) were determined by flow cytometry in the LM1 cell line before and after engraftment in the NOD-SCID mouse model. The ALCL cell line SU-DHL1 was used as positive control for CD30 and ALK expression while the BL cell line DG75 served as negative control.

**Figure 2:**
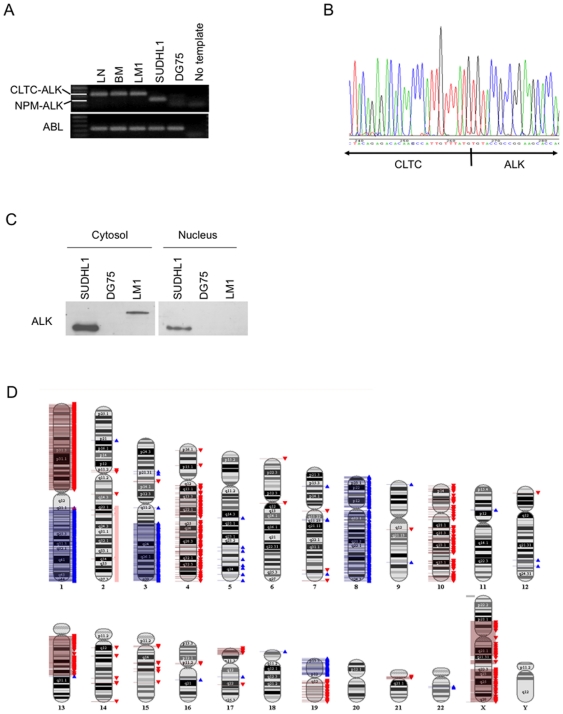
Genetic characterization of the LM1 cell line. **A**: The presence of the CLTC-ALK translocation in the patient's lymph node (LN), bone marrow infiltrating tumor cells (BM) and the established cell line (LM1) was determined with RT-PCR. The ALCL cell line SU-DHL1 was used as positive control for the NPM-ALK translocation. The BL cell line DG75 was used as negative control for both translocations involving ALK. ABL was used as loading control. **B**: Sequencing analysis indicated the presence of the CLTC-ALK fusion transcript in the LM1 cell line. **C**: Western blot for ALK showing exclusive cytoplasmic localization in the LM1 cell line corresponding to the expected molecular weight of the ALK-CLCT fusion protein. SU-DHL1 showed nuclear and cytosolic localization of Alk1 secondary to the NPM-ALK translocation. DG75 was used as negative control. **D**: SNP profiling of the LM1 cell line. Gains and losses are indicated in blue and red, respectively. Due to the nearly tetraploid chromosome status of this cell line the single loss of one copy in 2q22.1-qtel not detected by the software was determined manually (light red).

The cell line LM1 was established from the bone marrow of a 13 year-old girl suffering from a systemic relapse of a CLTC-ALK-positive DLBCL. The patient initially presented with a rapidly growing cervical and supraclavicular mass. Histopathological evaluation demonstrated large ALK-positive lymphoma cells suggestive of anaplastic large cell lymphoma of T- or 0-lineage (ALCL) and treatment was initiated accordingly [12]. The patient progressed locally after the first course of chemotherapy and an additional biopsy was taken. Revision of the histology of the initial biopsy as well as analysis of the second biopsy revealed the presence of ALK-positive DLBCL with expression of CD138, VS38c, CD38 and EMA, fine granular cytoplasmic ALK-staining and expression of the immunoglobulin kappa light chain as well as gamma heavy chain (**Figure 1**). Negativity for CD30, T cell markers (CD5, CD5, CD43, CD56, CD2, granzyme B) as well as CD20 and CD79a further confirmed the diagnosis. Molecular cytogenetics as well as RT-PCR for CLTC-ALK transcripts revealed t(2;17) (p23;q23) with expression of CLTC-ALK in the cells of the relapsed tumor (**Figure 2A**).

Despite subsequent intensive chemotherapy, the lymphoma progressed again locally. Highly intensive chemotherapy with autologous stem cell rescue and concomitant local radiotherapy was then administered, resulting in complete remission. This was followed by allogeneic blood stem cell transplantation. However, the patient relapsed 53 days later both locally and in the bone marrow. The infiltrating lymphoma cells were positive for CLTC-ALK, and were isolated for cell line derivation. These cells were kept under in vitro culture conditions using RPMI supplemented with penicillin/streptomycin, 4 mM L-glutamine and 20% fetal calf serum in a humidified incubator at 37°C with 5% CO_2_.

We determined the ability of these cells to propagate *in vitro* and whether they maintained the phenotype of the parental tumor. The immunophenotype of the cells in culture (from hereon named “LM1 cells”) was confirmed to be the same as the primary tumor: The cells expressed CD138, VS38c, CD38 and EMA, showed fine granular cytoplasmic ALK-staining and expression of the immunoglobulin kappa light chain as well as gamma heavy chain (**Figure 1**) Like the primary tumors, LM1 cells were negative for CD30, T cell markers, CD20 and CD79a (**Figure 1** and not shown). The expression of the CLTC-ALK fusion could be demonstrated by RT-PCR in both the primary tumor and in the LM1 cell line (**Figure 2A**). Sequencing analysis indicated the presence of the CLTC-ALK fusion transcript (**Figure 2B**). Immunoblot analysis with an Alk1 antibody showed exclusive cytoplasmic expressed protein of the expected molecular weight for CLTC-ALK (**Figure 2C**). The cell line carried a productively rearranged IGH sequence with a heavily mutated IGHV4-4 gene and a germline identity of only 86,61%.

The complex near-tetraploid karyotype of the cell line was: 74∼91<4n>,XXXX,del(1)(p10p35),t(2;17)(p23;q23)x2,add(2)(p11),der(4)t(4;15)(p14;q15),add(7)(q34∼35)x2,der(9)t(9;13)(q24;q12)x2,add(17)(p11)x2,inc[cp 15]. SNP analysis of mononuclear cells from the patient bone marrow and the established LM1 cell line detected a number of changes associated to the cell line including chromosomal gain in 1q, 3q13.31-qtel, 8, 11p13 and 19p as well as chromosomal loss in 1p, 2q22.1-qtel, 4q12-qtel, 7q36.3, 10, 13q11-q21.32, 13q21.33-q22.2, 17ptel-13p13.1, 17q22, 19q, and Xp21.1-q21.31, Xq21.33-q22.1, Xq22.3-qtel. No regions of partial uniparental disomy were identified (**Figure 2D** **and Table S2**). Moreover, 94.7% of the SNPs were identically called in the bone marrow normal mononuclear cells and in the derived cell line which, considering that imbalances reduce the numbers of identical calls, strongly supports the identity of the cell line.

### LM1 forms tumors in immunocompromised mice

To determine the ability of LM1 to grow in vivo, 1×10^7^ or 2×10^7^ cells were subcutaneously injected in the left flank of 10 SCID (5 per cell dosage) and 10 NOD-SCID (5 per cell dosage) mice. Between 16 and 28 days after the implantation, 3/10 and 9/10 mice grew tumors in the SCID and NOD-SCID background, respectively. The NOD-SCID mouse was considered the most appropriate host and 1×10^7^ cells were xenografted in subsequent experiments. We evaluated the characteristics of the LM1 tumor mass comparing them to the primary tumor as well as to the LM1 cell line. In concordance with the original tumor and the LM1 cell line, the LM1 xenograft revealed the presence of plasmoblastic DLBCL with expression of fine granular cytoplasmic ALK-staining, expression of the immunoglobulin kappa light chain, CD138 and negativity for CD30 (**Figure 1**), indicating that the cellular features were maintained in the xenografted tumor. Taken together, these data suggest that the LM1 cell line is an adequate model to study the biology and therapeutic targeting of ALK fusion positive DLBCL.

### ALK kinase inhibition induces cell death in LM1 cells *in vitro*

**Figure 3:**
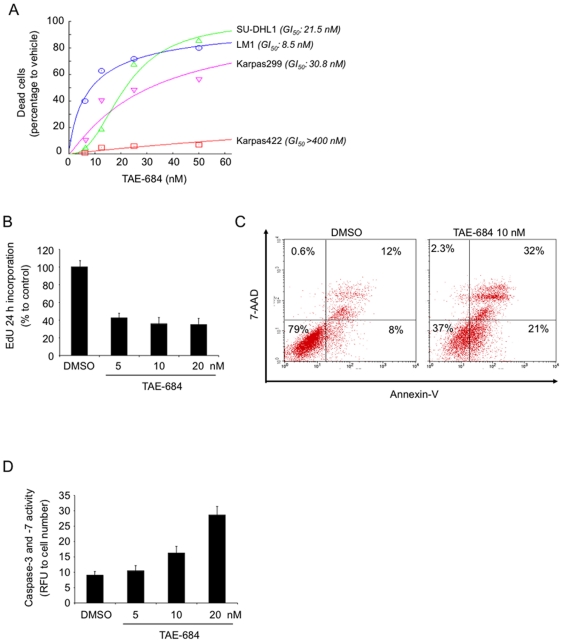
In vitro effect of the ALK inhibitor TAE-684. **A**: The cell lines SU-DHL1 (ALCL), Karpas299 (ALCL) and LM1 (DLBCL) harboring ALK translocations and the cell line Karpas422 (as negative control) were exposed in six replicates to 8 concentrations (from 1 to 400 nM) of the ALK inhibitor TAE-684 or vehicle control (DMSO) for 48 h and analyzed for viability. Dose-response curves were plotted. The X-axis shows the dose of TAE-684 in nM. The Y-axis shows the effect of TAE-684 as compared to vehicle control on cell viability. The experiment was done in triplicates. The goodness of fit for the experimental data to the median-effect equation (linear correlation coefficient) obtained from the logarithmic form of this equation was equal or higher than 0.90 for each curve. The concentration of TAE-684 that inhibits 50% the growth of cell lines compared to control treated cells (GI_50_) is shown in parentheses. **B**: Cell proliferation determined by EdU incorporation in DMSO- and TAE-684-treated cells (X-axis, concentration in nM). Results are expressed as percentage to control after normalization to viable cells. **C**: Apoptosis in LM1 cells measured by Annexin-V (and 7-AAD for nuclear staining) after incubation with DMSO or TAE-684 10 nM for 24 h (one representative of triplicate experiments is shown). **D**: Caspase-3 and -7 activity in LM1 cells determined after 24 h of exposure to DMSO- or TAE-684- at several concentration (X-axis). Results are expressed in relative fluorescent units (Y-axis) normalized to cell number.

The selective ALK inhibitor TAE-684 was shown to have activity against NPM-ALK positive ALCL cell lines *in vitro* and *in vivo* [10]. In order to determine whether an ALK inhibitor also had activity in CLTC-ALK positive DLBCL, we exposed LM1 cells to increasing concentrations of TAE-684. The NPM-ALK positive ALCL cell lines Karpas299 and SUDHL1 were used as positive controls [10] while the ALK negative DLBCL cell line Karpas422 served as negative control. In agreement with previous publications [10], SUDHL1 and Karpas299 were susceptible to TAE-684 (GI_50_ of 21.5 and 30.8 nM respectively) while Karpas422 was resistant (**Figure 3A**). TAE-684 inhibited the growth of LM1 at low nanomolar concentrations (GI_50_: 8.5 nM) (**Figure 3A**).

To further characterize the biological effects of ALK inhibition on the growth and survival of the LM1 cell line, we performed proliferation, cell cycle and apoptosis analysis on cells treated with either TAE-684 or DMSO control. LM1 cells were treated with increasing concentrations of TAE-684 (DMSO, 5, 10 and 20 nM) for 24 h and assessed for proliferation by a nucleoside analog (EdU) DNA incorporation assay. Treatment with TAE-684 decreased the EdU incorporation in LM1 cells (**Fig. 3B**) indicating that exposure to TAE-684 inhibited proliferation. Since different NPM-ALK positive ALCL cell lines have been reported to respond differentially with either apoptosis or G1-cell cycle arrest [10], we wished to determined whether the effect on proliferation was due to preferential cell cycle arrest, cell death or a combination of both. We analyzed cell cycle distribution by flow cytometry DNA deconvolution at 4, 12 and 24 h after treatment. TAE-684 10 nM caused G1 cell cycle arrest at 24 h in Karpas299 cells but not in LM1 (**Figure S1**). There was no cell cycle arrest in LM1 at any of time points analyzed, suggesting that cell death is the main mechanism for growth inhibition in this cell line. Accordingly, TAE-684 exposure for 24 h induced apoptosis in a dose-dependent manner in LM1 cells as detected by Annexin-V staining (**Figure 3C**) and caspase-7 and -3 activation (**Figure 3D**). Apoptosis induction was morphologically confirmed with ethidium bromide and orange G staining under fluorescence microscopy. Collectively, these data suggest that inhibition of ALK kinase activity by TAE-684 reduces the growth of LM1 cells by preferentially inducing apoptosis.

### TAE-684 inhibits signalling downstream of CLTC-ALK

**Figure 4:**
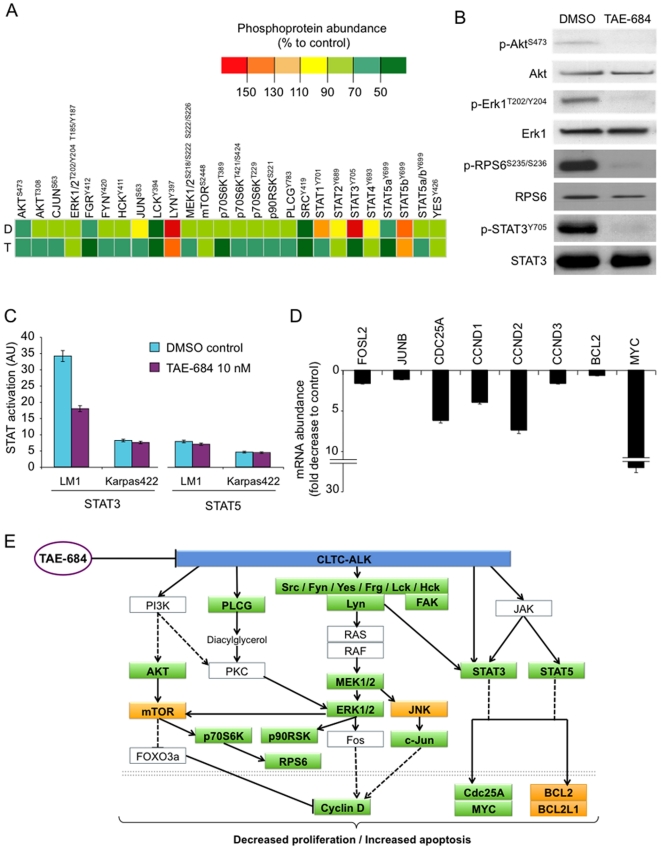
TAE-684 inhibits signalling downstream of CLTC-ALK in LM1 cells. **A**: Phosphoprotein abundance in LM1 cells treated with DMSO (D) or TAE-684 10 nM for 4 h (T). The heat map represent the relative abundance normalized by the positive control in the proteome array (see also **Supp. Fig. 2**). The particular protein residue phosphorylated is shown as superscript. **B**: Western blot for phospho-ALK, ALK, phospho-ERK1, ERK1, phospho-RPS6, RPS6, phospho-STAT3 and STAT3 in cytosol lysates of LM1 cells treated with DMSO or TAE-684 10 nM for 4 h. **C**: STAT 3 and STAT5 DNA binding activity (Y-axis, arbitrary units, AU) in Karpas422 and LM1 cells treated with DMSO (representing the baseline activity) and after 4 h of exposure to 10 nM of TAE-684. Experiments represent duplicates with SEM. **D**: Transcript abundance for FOSL2, JUNB, CDC25A, CCND1, CCND2, CCND3, BCL2 and MYC in LM1 cells treated with DMSO (control) or TAE-684 10 nM for 12 h. Data was normalized to RPL13A levels. Data is presented as fold decrease compared to control (DMSO treated cells). **E**: Signaling pathways modified in LM1 cells by treatment with TAE-684. Phosphoproteins (transcripts are represented bellow the double dotted line) with significant decreases by TAE-684 are shown in green boxes, with no changes in yellow boxes and those not determined in white boxes.

Fusions of ALK have oncogenic potential as its aberrant kinase activity enhances cell proliferation and survival [3]. Similarly to most normal and oncogenic tyrosine kinases, ALK fusions activate many interconnected and redundant pathways. The most relevant and characterized pathways are the ERK, JAK-STAT3 and PI3K-AKT pathways [3], [13]. To determine what pathways are preferentially affected with TAE-684 in LM1 cells, we performed a phosphoprotein array in these cells treated with DMSO and TAE-684 at 10 nM for 24 h. The most affected protein in the array was STAT3 (**Figure 4A** **and Figure S2**). STAT3 phosphorylation in tyrosine 705 decreases 5 fold after TAE-684 (**Figure 4A**). Additional proteins with significant decreases were: p70S6K^T389^ (3.2 fold), STAT1^Y701^ (2.6 fold), FAK^Y397^ (2.4 fold), LCK^Y394^ (2 fold) and STAT5a/b^Y699^ (2 fold) (**Figure 4A** **and Figure S2**). There were more modest reductions in the phosphorylation of other proteins such as p90RSK, ERK1/2, AKT, c-JUN, STAT1, STAT2 and several members of the SRC family (LYN, HCK, FYN, FRG) among others. We validated some of these changes in an independent experiment using immunoblots (**Figure 4B**). In addition to changes in AKT, ERK1 and STAT3 phosphorylation following TAE-684 treatment, we found a decrease in phospho-RPS6^S235/S236^, a protein not included in the array (**Figure 4B**). In contrast to STAT3 [3], the role of STAT5 in ALK fusion-mediated lymphomagenesis is more controversial. [3], [14]. To determine whether STAT3 or STAT5 signalling are functional in CLTC-ALK in DLBCL, we performed DNA binding assays on lysates of LM1 and Karpas422 cells treated with DMSO or TAE-684 10 nM for 4 h. In concordance with the protein levels, the baseline activity of STAT3 was higher in LM1 compared to Karpas422 cells (**Figure 4C**), as determined by the respective DNA binding capacity, whereas the DNA-binding of STAT5 was only slightly higher in LM1 compared to Karpas422 (**Figure 4C**). After 4 h of treatment with TAE-684 10 nM, STAT3 activity levels decreased significantly (37.1±1.8 AU to 20.9±1 AU, p<0.001) in LM1 cells, but not in Karpas442 cells (8.2±1.6 AU to 7.5±1.2 AU) (**Figure 4C**). In contrast, the activity of STAT5 did not change significantly after TAE-684 in either cell line (**Figure 4C**). The impact of CLTC-ALK inhibition on the cellular transcriptional activity was determined by the mRNA abundance of several target genes related to these pathways. In LM1 cells treated with TAE-684 10 nM for 12 h, we found a decrease in FOSL2, JUNB, CDC25A, CCND1, CCND2, CCND3, BCL2 and MYC (**Figure 4D**) transcript abundance. Other target genes related to these pathways (BCL2L2, MCL1, CDKN1A, FOS, FOSB, FOSL1, JUN and JUND) did not change significantly under the experimental conditions (data not shown). The changes in the CLTC-ALK related pathways with TAE-684 treatment, including those in phosphoprotein levels and mRNA abundance, are summarized in **Figure 4E**. Taken together, our data suggest that constitutive ALK activity of CLTC-ALK fusion proteins induces similar survival and proliferative signalling cascades in DLBCL as NPM-ALK in ALCL.

### TAE-684 induces complete regression of LM1 xenografts

**Figure 5:**
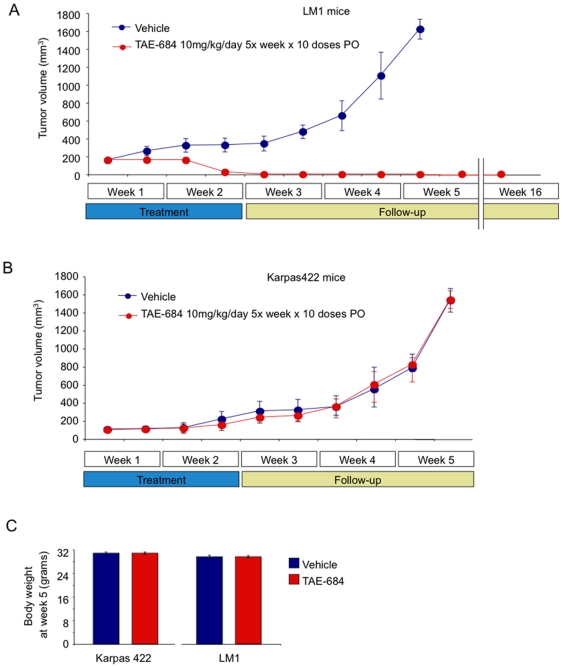
Effect of the ALK inhibitor TAE-684 on the growth of LM1 and Karpas422 xenografts. **A**: Tumor growth plot in LM1 xenografted mice treated with vehicle (blue circles) or TAE-684 at 10 mg/kg/day 5 days per week for 2 weeks (red circles). The mice were followed without treatment until the tumors reached 1500 mm^3^ when the mice were killed (beginning of week 5). The treated mice were followed without further treatment for a total of 16 weeks when they were also killed. The treated mice had no macro or microscopic evidence of tumor relapse. The Y-axis indicates tumor volume (in mm^3^) and X-axis days of treatment. **B**: The same experiment as in panel A but using Karpas422 mice. In this case all the mice were killed when the tumors reached 1500 mm^3^ (at the end of week 5). **C**: Adjusted body weight at week 5 in the Karpas422 and LM1 mice for the control and TAE-684-treated animals. The error bars represent the SEM.

In order to evaluate the anti-lymphoma activity of TAE-684 *in vivo*, the LM1 cell line was injected into the right flank of 10 NOD-SCID mice and allowed to form tumors. Once palpable tumors were detected, pairs of mice were randomized to receive either TAE-684 10 mg/kg/day 5 days per week for 2 weeks (n = 5) or vehicle (n = 5). The drug and vehicle were administered by oral gavage. The ALK fusion negative DLBCL cell line Karpas422 was also implanted in NOD-SCID mice (n = 10) and treated in the same way. TAE-684 induced regression of the LM1 tumors by the second week and complete remission by the third week (**Figure 5A**). Remission was sustained without recurrence of tumors in any of the animals for 13 additional weeks after which the experiment was terminated and the animals sacrificed (**Figure 5A**). In contrast, Karpas422 xenografted tumors were unaffected by the drug and grew at the same rate as vehicle controls (**Figure 5B**). In both models, macroscopic and microscopic examination of the animals showed no signs of disease or organ toxicity. The adjusted body weight between treated and controls were similar for Karpas422 and LM1 animals (**Figure 5C**).

## Discussion

Here we describe the establishment of the first CLTC-ALK positive DLBCL cell line from the bone marrow of a patient with chemotherapy-resistant lymphoma. This cell line, LM1, carries the same phenotypic and genotypic characteristics as the malignant cells from the subject. LM1 forms tumors in mice with a similar growth pattern compared to other established mouse xenograft models of human DLBCL. LM1 can thus serve as pre-clinical testing platform for the role of CLTC-ALK in lymphomagenesis and developing molecular targeted therapy approaches for CLTC-ALK positive DLBCL.

Our data gained from pharmacological inhibition of ALK-activity *in vitro* and *in vivo* suggest that CLTC-ALK mediates DLBCL lymphomagenesis and maintenance by constitutive ALK kinase activity. This observation is in line with data indicating that CLTC-ALK transforms fibroblasts as efficiently as other ALK-fusion proteins [15]. Additionally, our data lend further support to the notion that ALK-fusion proteins confer high oncogenic potential to transformed cells of different origin independently of the fusion partner and induce both B- and T-cell lymphomas in transgenic mice [3], [16].

Several small molecule kinase inhibitors have been developed blocking ALK-kinase activity and signal transduction in a concentration-dependent manner [10], [18], [19]. This development opens the possibility of targeted therapy for ALK-positive malignancies. Patients with ALK-positive ALCL have a good overall survival due, in part, to effective relapse strategies including immunotherapeutic approaches [17], [18]. In contrast to ALCL, the available reports suggest that ALK-positive DLBCL is often a chemorefractory disease associated with a poorer outcome [8], [9]. These patients might, therefore, be candidates for clinical trials with ALK-inhibitors. The high *in vitro* and *in vivo* sensitivity of LM1 cells to ALK inhibition supports the rationale testing these compounds for ALK-positive DLBCLs.

NPM-ALK-positive cells show activation of signaling pathways, such as Src kinases [19], PI3K-AKT [20], ERK [21] and STAT3 and 5 [13], [14], [22]. Functional studies suggest a pivotal role of STAT3 and the PI3K-AKT pathway in NPM-ALK mediated lymphomagenesis whereas a role for STAT5 is more controversial [13],[14],[22]. While ectopic expression of CLTC-ALK in fibroblasts induced less STAT3 phosphorylation than other ALK-fusion proteins [15], a recent immunohistological study detected ubiquitous STAT3 hyperphosphorylation in two CLTC-ALK positive DLBCL cases compared to ALK negative DLBCL [23]. In our study CLTC-ALK positive DLBCL cells exhibited constitutive STAT3 activity as well as activation of Akt and ERK. Inhibition of ALK-activity decreased the activity of these three signaling pathways in LM1 cells suggesting that CLTC-ALK employs similar signaling cascades than NPM-ALK.

Taken together, our data demonstrate that LM1 is a *bona fide* model of the DLBCL subtype featuring the CLTC-ALK translocation and indicate that growth of CLTC-ALK positive DLBCL is dependent on ALK kinase. Patients diagnosed with ALK positive DLBCL may, therefore, be candidates for therapeutic trials of ALK inhibitors. The incorporation of ALK status determination into the histopathological characterization of DLBCL could help identifying these patients more readily.

## Supporting Information

Figure S1 **Cell cycle analysis.** LM1 and Karpas299 (positive control) cells were assessed for cell cycle distribution by propidium iodide staining and flow cytometry after treatment with TAE-684 10 nM or DMSO for 24 h. One representative experiment from triplicates is shown.
(TIF)

Figure S2 **Phosphoprotein array.** Scanned image of the phosphoprotein array in LM1 cells treated with DMSO (left) or TAE-684 10 nM for 4 h (right). Certain proteins of interest with the correspondent phosphorilated residue are identified. A complete map of the array can be found at http://www.rndsystems.com/pdf/ary003.pdf
(TIF)

Table S1 **Additional RT-PCR primers.**
(TIF)

Table S2 **Chromosomal gains and losses in the LM1 cell line.** Chromosomal gain and losses were determined by SNP array. The cytoband and linear position (bp) are in accordance to the NCBI Build 36.1.
(TIF)
