# Supplementary figures and images for: Inhibition of Anaplastic Lymphoma Kinase (ALK) Activity Provides a Therapeutic Approach for CLTC-ALK-Positive Human Diffuse Large B Cell Lymphomas
Source: PLoS One. 2011 Apr 8;6(4):e18436. doi: 10.1371/journal.pone.0018436 (PMC3072987; doi:10.1371/journal.pone.0018436)

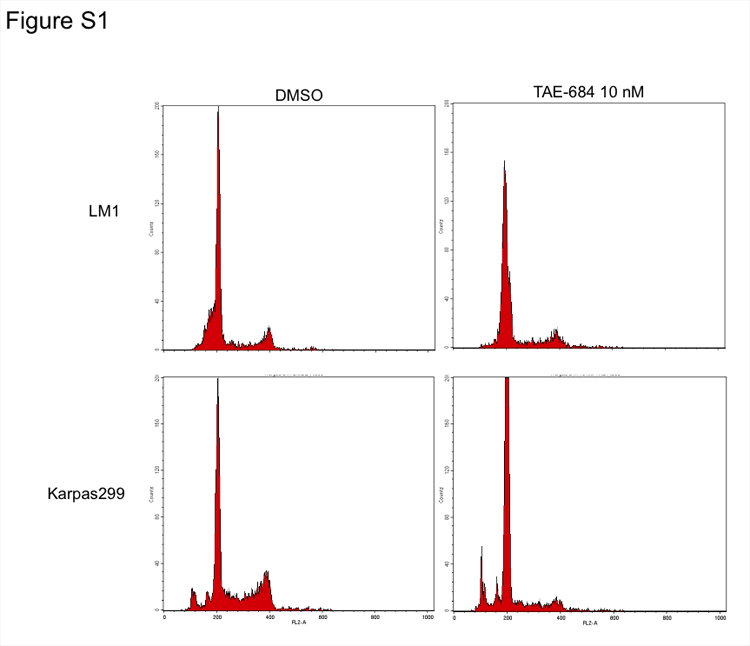

Supplement: Figure S1 — Cell cycle analysis. LM1 and Karpas299 (positive control) cells were assessed for cell cycle distribution by propidium iodide staining and flow cytometry after treatment with TAE-684 10 nM or DMSO for 24 h. One representative experiment from triplicates is shown. (TIF) [file pone.0018436.s001.tif]

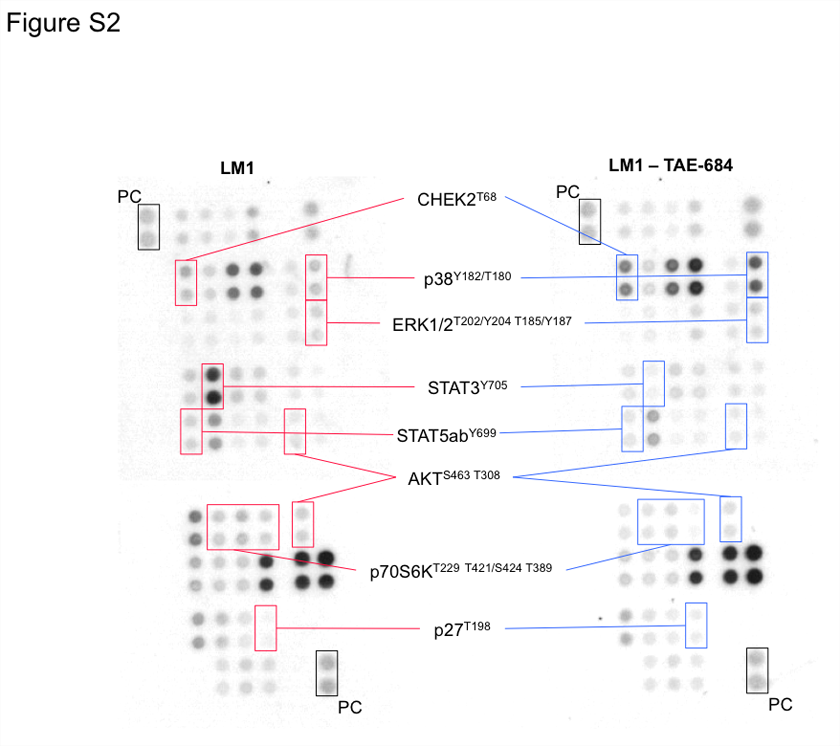

Supplement: Figure S2 — Phosphoprotein array. Scanned image of the phosphoprotein array in LM1 cells treated with DMSO (left) or TAE-684 10 nM for 4 h (right). Certain proteins of interest with the correspondent phosphorilated residue are identified. A complete map of the array can be found at http://www.rndsystems.com/pdf/ary003.pdf (TIF) [file pone.0018436.s002.tif]

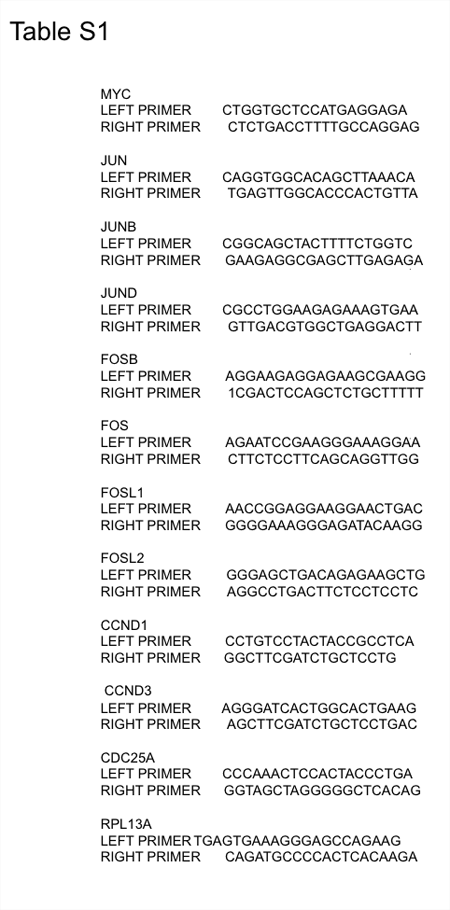

Supplement: Table S1 — Additional RT-PCR primers. (TIF) [file pone.0018436.s003.tif]

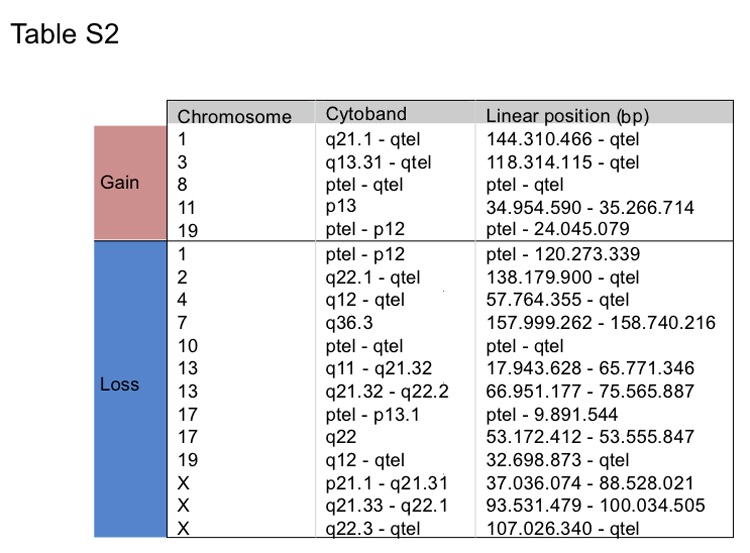

Supplement: Table S2 — Chromosomal gains and losses in the LM1 cell line. Chromosomal gain and losses were determined by SNP array. The cytoband and linear position (bp) are in accordance to the NCBI Build 36.1. (TIF) [file pone.0018436.s004.tif]
